# *Spica prunellae* promotes cancer cell apoptosis, inhibits cell proliferation and tumor angiogenesis in a mouse model of colorectal cancer via suppression of stat3 pathway

**DOI:** 10.1186/1472-6882-13-144

**Published:** 2013-06-24

**Authors:** Wei Lin, Liangpu Zheng, Qunchuan Zhuang, Jinyan Zhao, Zhiyun Cao, Jianwei Zeng, Shan Lin, Wei Xu, Jun Peng

**Affiliations:** 1 Academy of Integrative Medicine, Fujian University of Traditional Chinese Medicine, 1 Huatuo Road, Minhou Shangjie, Fuzhou, Fujian 350122, China; 2 Fujian Key Laboratory of Integrative Medicine on Geriatrics, Fujian University of Traditional Chinese Medicine, 1 Huatuo Road, Minhou Shangjie, Fuzhou, Fujian 350122, China; 3 Department of Pharmacology, Fujian University of Traditional Chinese Medicine, 1 Huatuo Road, Minhou Shangjie, Fuzhou, Fujian 350122, China

**Keywords:** *Spica prunellae*, Colorectal cancer, Herbal medicine, STAT3 pathway, Apoptosis, Proliferation, Angiogenesis

## Abstract

**Background:**

Constitutive activation of STAT3 is one of the major oncogenic pathways involved in the development of various types of malignancies including colorectal cancer (CRC); and thus becomes a promising therapeutic target*. Spica Prunellae* has long been used as an important component in many traditional Chinese medicine formulas to clinically treat CRC. Previously, we found that *Spica Prunellae* inhibits CRC cell growth through mitochondrion-mediated apoptosis. Furthermore, we demonstrated its anti-angiogenic activities *in vivo* and *in vitro*. To further elucidate the precise mechanism of the potential tumoricidal activity of *Spica Prunellae*, using a CRC mouse xenograft model, in this study we evaluated its therapeutic efficacy against CRC and investigated the underlying molecular mechanisms.

**Methods:**

CRC mouse xenograft model was generated by subcutaneous injection of human colon carcinoma HT-29 cells into nude mice. Animals were given intra-gastric administration with 6 g/kg of the ethanol extract of *Spica Prunellae* (EESP) daily, 5 days a week for 16 days. Body weight and tumor growth were measured every two days. Tumor growth *in vivo* was determined by measuring the tumor volume and weight. HT-29 cell viability was examined by MTT assay. Cell apoptosis and proliferation in tumors from CRC xenograft mice was evaluated via immunohistochemical staining (IHS) for TUNEL and PCNA, and the intratumoral microvessel density (MVD) was examined by using IHS for the endothelial cell-specific marker CD31. The activation of STAT3 was evaluated by determining its phosphorylation level using IHS. The mRNA and protein expression of Bcl-2, Bax, Cyclin D1, VEGF-A and VEGFR2 was measured by RT-PCR and IHS, respectively.

**Results:**

EESP treatment reduced tumor volume and tumor weight but had no effect on body weight change in CRC mice; decreased HT-29 cell viability in a dose-dependent manner, suggesting that EESP displays therapeutic efficacy against colon cancer growth *in vivo* and *in vitro*, without apparent toxicity. In addition, EESP significantly inhibited the phosphorylation of STAT3 in tumor tissues, indicating its suppressive action on the activation of STAT3 signaling. Consequently, the inhibitory effect of EESP on STAT3 activation resulted in an increase in the pro-apoptotic Bax/Bcl-2 ratio, decrease in the expression of the pro-proliferative Cyclin D1 and CDK4, as well as down-regulation of pro-angiogenic VEGF-A and VEGFR-2 expression. Finally, these molecular effects led to the induction of apoptosis, the inhibition of cell proliferation and tumor angiogenesis.

**Conclusions:**

*Spica Prunellae* possesses a broad range of anti-cancer activities due to its ability to affect STAT3 pathway, suggesting that *Spica Prunellae* could be a novel potent therapeutic agent for the treatment of CRC.

## Background

Colorectal carcinoma (CRC) is a serious public health problem, with over one million new cases worldwide each year [1]. In Western societies CRC represents the second most common cause of cancer-related death [2]. The pathogenesis of CRC is complex and heterogeneous, with the involvement of multiple cellular signaling transduction cascades including signal transducer and activator of transcription 3 (STAT3). As one of the most important transcription factors, STAT3 is involved in the control of many fundamental biological processes, such as cell proliferation, apoptosis and angiogenesis [3-8]. STAT3 can be activated by many cytokines and growth factors, such as IL-6 and EGF [9,10], which is mediated by phosphorylation of STAT3 at tyrosine 705 after the binding of IL-6 or EGF to the specific cell surface receptors [11]. Phosphorylated STAT3 proteins in the cytoplasm dimerize and translocate to the nucleus where they regulate the expression of genes containing STAT3-binding sites in their promoters [12]. STAT3 activation is rapid and transient in normal cells; however, in cancer cells STAT3 is constitutively activated, resulting in imbalance between cell proliferation and apoptosis, and uncontrolled tumor angiogenesis. Constitutive activation of STAT3 has been found in numerous types of human cancer including CRC and commonly suggests poor prognosis [13-23].Therefore, suppression of STAT3 pathway has been a major therapeutic target for treatment of cancer.

Despite recent advances in CRC chemotherapy, 5-fluorouracil (5-FU)-based regimens continue to be the international standard chemotherapy for patients with invasive and metastatic advanced CRC [24]. However, due to drug resistance and the unacceptable level of toxicity to normal cells, systemic chemotherapy using 5-FU-based regimens produces objective response rates of less than 40% [25,26]. These problems highlight the urgent need for the development of novel cancer chemotherapies. Natural products, such as traditional Chinese herbal medicines, have received attention as they have relatively few side-effects and have long been used clinically as significant alternative remedies for a variety of diseases [27-29]. *Spica Prunellae*, the fruit-spikes of the perennial plant *Prunella vulgaris L*., is a medicinal herb widely distributed in northeast Asia. As a well-known Chinese folk medicinal herb with properties of heat-clearing and detoxification, *Spica Prunellae* is used to treat poor vision, blood stasis and edema, acute conjunctivitis, lymphatic tuberculosis and scrofula, acute mastitis and mammary gland hyperplasia, thyromegaly, and hypertention [30]. Moreover, *Spica Prunellae* is believed to possess anti-cancer activity since in traditional Chinese medicine (TCM) system, accumulation of heat and toxic dampness is a major causative factor for tumorigenesis. Indeed, *Spica Prunellae* has long been used as an important component in several TCM formulas for the clinical treatment of several kinds of cancer including CRC [31,32]. Although we previously reported that the extract of *Spica Prunellae* promotes the apoptosis of human colon carcinoma cells and displays anti-angiogenic activity *in vitro*[33,34], the mode of its anti-cancer action remains largely unknown. To further elucidate the mechanism of the tumorcidal activity of *Spica Prunellae*, using a CRC mouse xenograft model in the present study we evaluated the therapeutic efficacy of the ethanol extract of *Spica Prunellae* (EESP) against tumor growth *in vivo* and investigated the underlying molecular mechanisms.

## Methods

### Materials and reagents

Dulbecco’s modified Eagle’s medium (DMEM), fetal bovine serum (FBS), penicillin-streptomycin, trypsin-EDTA, Trizol reagent were purchased from Invitrogen (Carlsbad, NM, USA). SuperScript II reverse transcriptase was provided by Promega (Madison, WI, USA). PCNA, CD31, Bcl-2, Bax, CyclinD1, CDK4, VEGF-A, VEGFR antibodies, horseradish peroxidase (HRP)-conjugated secondary anti-bodies were obtained from Cell Signaling (Beverly, MA, USA). TUNEL assay kit was purchased from R&D Systems (Minneapolis, MN, USA). All the other chemicals used, unless otherwise stated, were obtained from Sigma Chemicals (St. Louis, MO, USA).

### Preparation of ethanol extract from *spica prunellae*

Authentic plant material was purchased from Guo Yi Tang Chinese Herbal medicine store (Fujian, China). The original herb was collected in Hunan Province, China, and was identified as *Spica Prunellae* by Professor Chengzi Yang at Department of Pharmacology, Fujian University of Traditional Chinese Medicine (FJTCM), China. Voucher specimen was deposited at the Herbarium of the Department of Pharmacology, FJTCM. 500 g of *Spica Prunellae* were extracted with 5000 ml of 85% ethanol using a refluxing method and filtered. The ethanol solvent was evaporated on a rotary evaporator (Shanghai Yarong, Model RE-2000, China) and concentrated to a relative density of 1.05. A dried powder of ethanol extract of *Spica Prunellae* (named EESP) was obtained by spray desiccation using a spray dryer (Buchi, Model B-290, Swiss). For animal experiments, the powder of EESP was dissolved in saline to a working concentration of 600 mg/ml. The stock solution of EESP in cell-based experiments was prepared by dissolving EESP powder in DMSO to a stock concentration of 200 mg/ml and the working concentrations were made by diluting the stock solution in the cell culture medium. The final concentration of DMSO in the medium for all cell experiments was < 0.5%.

### HPLC analysis

**Figure 1:**
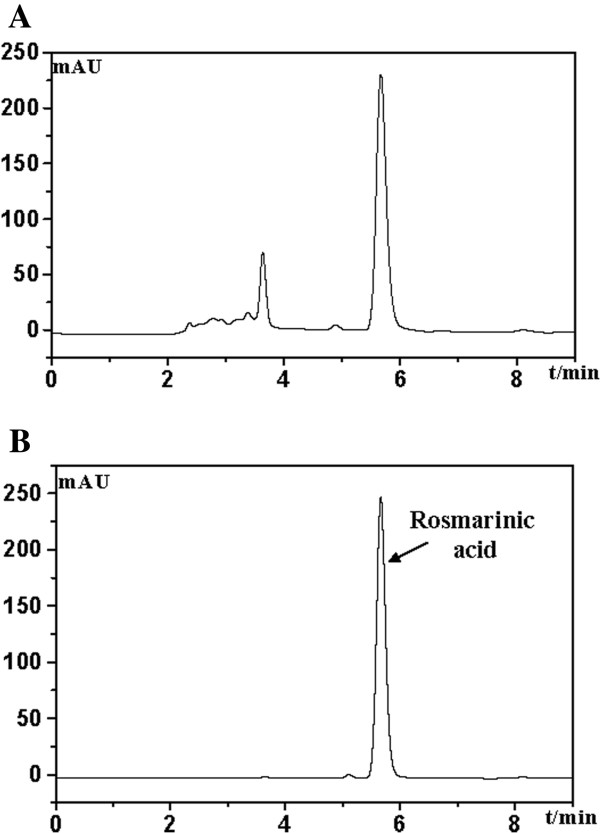
**HPLC analysis of EESP.** The mobile phase consisted of methanol:0.1% phosphoric acid solution (50:50) at a flow rate of 1 ml/min. (**A**) EESP; (**B**) Rosmarinic acid.

EESP was analyzed on an Agilent 1200 HPLC system (Agilent, Santa Clara, CA, USA) using a C-18 column (4.6 mm × 150 mm, 5 μm). Absorbance was measured at 355 nm (Figure 1). The mobile phase consisted of methanol: 0.1% phosphoric acid solution (50:50) at a flow rate of 1 ml/min with an injection volume of 10 μl. A sample containing Rosmarinic acid was used as a control.

### Cell culture

Human colon carcinoma HT-29 cells were obtained from American Type Culture Collection (ATCC, Manassas, VA, USA). The cells were grown in DMEM containing 10% (v/v) FBS, and 100 Units/ml penicillin and 100 μg/ml streptomycin in a 37°C humidified incubator with 5% CO_2_. The cells were subcultured at 80-90% confluency.

### Animals

Male BALB/c athymic (nude) mice (with an initial body weight of 20–22 g) were obtained from Shanghai SLAC Laboratory Animal Co., Ltd. (Shanghai, China) and housed under pathogen-free conditions with controlled temperature (22°C), humidity, and a 12 hour light/dark cycle. Food and water were given *ad libitum* throughout the experiment. All animal treatments were performed strictly in accordance with international ethical guidelines and the National Institutes of Health Guide concerning the Care and Use of Laboratory Animals. The experiments were approved by the Institutional Animal Care and Use Committee of Fujian University of Traditional Chinese Medicine.

### In vivo tumor xenograft study

HT-29 cells were grown in culture and then detached by trypsinization, washed, and resuspended in serum-free DMEM. 1.5 × 10^6^ of cells mixed with Matrigel (1:1) were subcutaneously injected in the right flank area of athymic nude mice to initiate tumor growth. After 3 days of xenograft implantation, mice were randomized into two groups (*n* = 9) and given intra-gastric administration of 6 g/kg of EESP or saline daily, 5 days a week for 16 days. Body weight and tumor growth were measured every two days. Tumor growth was determined by measuring the major (L) and minor (W) diameter with a caliper. The tumor volume was calculated according to the following formula: tumor volume = π/6 × L × W^2^. At the end of experiment, the animals were anaesthetized with pelltobarbitalum natricum, and the tumor tissue was removed and weighed, and part of tumor was fixed in buffered formalin and the remaining was stored at -80°C for molecular analyses.

### Evaluation of cell viability by MTT Assay

Cell viability was assessed by the 3-(4, 5-dimethylthiazol-2-yl)-2, 5-diphenyltetrazolium bromide (MTT) colorimetric assay. HT-29 cells were seeded into 96-well plates at a density of 1 × 10^4^ cells/well in 0.1 ml medium. The cells were treated with various concentrations of EESP for 24 h. Treatment with 0.5% DMSO was included as vehicle control. At the end of the treatment, 10 μl MTT (5 mg/ml in phosphate buffered saline, PBS) were added to each well, and the samples were incubated for an additional 4 h at 37°C. The purple-blue MTT formazan precipitate was dissolved in 100 μl DMSO. The absorbance was measured at 570 nm using an ELISA reader (BioTek, Model ELX800, USA).

### Histological examination by HE staining

Tumor tissues were fixed with 10% buffered formalin for 24 h. Samples were then paraffin-embedded, sectioned, and stained with hematoxylin and eosin (H&E). Histopathological changes were observed under a light microscope.

### RNA extraction and RT-PCR analysis

Briefly, Total RNA was isolated from fresh tumor with Trizol reagent. Oligo(dT)-primed RNA (1 μg) was reverse transcribed with SuperScript II reverse transcriptase according to the manufacturer’s instructions. The obtained cDNA was used to determine the mRNA amount of Bcl-2, Bax, Cyclin D1, CDK4, VEGF-A and VEGFR2 by PCR with Taq DNA polymerase (Fermentas). GAPDH was used as an internal control. The primers used for amplification of Bcl-2, Bax, Cyclin D1, CDK4, VEGF-A , VEGFR2, and GAPDH transcripts are as follows: Bcl-2 forward 5′-CAG CTG CAC CTG ACG CCC TT-3 and reverse 5′-GCC TCC GTT ATC CTG GAT CC-3′; Bax forward 5′-TGC TTC AGG GTT TCA TCC AGG-3′ and reverse 5′-TGG CAA AGT AGA AAA GGG CGA-3′; Cyclin D1 forward 5′-TGG ATG CTG GAG GTC TGC GAG GAA-3′ and reverse 5′-GGC TTC GAT CTG CTC CTG GCA GGC-3′; CDK4 forward 5′-CAT GTA GAC CAG GAC CTA AGC-3′ and reverse 5′-AAC TGG CGC ATC AGA TCC TAG-3′; GAPDH forward 5′-GT CAT CCA TGA CAA CTT TGG-3′ and reverse 5′-GA GCT TGA CAA AGT GGT CGT-3′; VEGF-A forward 5- CAT CCT GGC CTC GCT GTC −3 and reverse 5- CTC GCT CCA ACC GAC TGC −3; VEGFR-2 forward 5- TGG CTC ACA GGC AAC ATC −3 and reverse 5- CTT CCT TCC TCA CCC TTC −3.

### In situ apoptosis detection by TUNEL

Briefly, sequential 4 μm tissue sections were adhered to silane-coated slides and allowed to dry at room temperature (RT). Subsequently, sections were deparaffinized and rehydrated. Protein digestion was done by incubating tissue sections in 20 mg/ml proteinase K (Worthington Co., Lakewood, USA) for 15 min at RT. Endogenous peroxidase was inactivated with 2% H_2_O_2_ in distilled water (dH_2_O) for 5 min, RT. The labeling mixture containing biotinylated dUTP in TdT enzyme buffer was added to sections and incubated at 37°C in a humified chamber for 1 h. After stopping the enzymatic reaction, sections were rinsed with PBS, covered with anti-digoxigenin peroxidase conjugate and incubated for 30 min at RT in a humified chamber. Then, sections were incubated in TBS with 0.05% diaminobenzidine (DAB) plus 3% H_2_O_2_ until color development achieved. Finally, sections were washed, counterstained in haematoxylin, dehydrated, and mounted with DPX (Panreac SA, Barcelona, Spain) and as a negative control active TdT buffer was replaced by the kit equilibration buffer.

### Immunohistochemostry

After fixed with 10% formaldehyde for 12 h, tumor samples were processed conventionally for paraffin-embedded tumor slides. The slides were subjected to antigen retrieval and the endogenous peroxidase activity was quenched with hydrogen peroxide. After blocking non-specific proteins with normal serum in PBS (0.1% Tween 20), slides were incubated with rabbit polyclonal antibodies against PCNA, CD31, pSTAT3, Bcl-2, Bax, CyclinD1, CDK4, VEGF-A and VEGFR-2 (all in 1:200 dilution). After washing with PBS, slides were incubated with biotinylated secondary antibody followed by conjugated horseradish peroxidase (HRP)-labelled streptavidin (Dako), and then washed with PBS. The slides were then incubated with diamino-benzidine (DAB, Sigma) as the chromogen, followed by counterstaining with diluted Harris hematoxylin (Sigma). After staining, five high-power fields (400 ×) were randomly selected in each slide, and the average proportion of positive cells in each field were counted using the true color multi-functional cell image analysis management system (Image-Pro Plus, Media Cybernetics, USA). To rule out any nonspecific staining, PBS was used to replace the primary antibody as a negative control.

### Statistical analysis

Data were presented as mean ± SD for the indicated number of independently performed experiments and the data were analyzed using the SPSS package for Windows (Version 11.5). Statistical analysis of the data was performed with the Student’s t-test. Differences with *P* < 0.05 were considered statistically significant.

## Results

### EESP inhibits the growth of colorectal cancer (CRC) *in vivo* and *in vitro*

**Figure 2:**
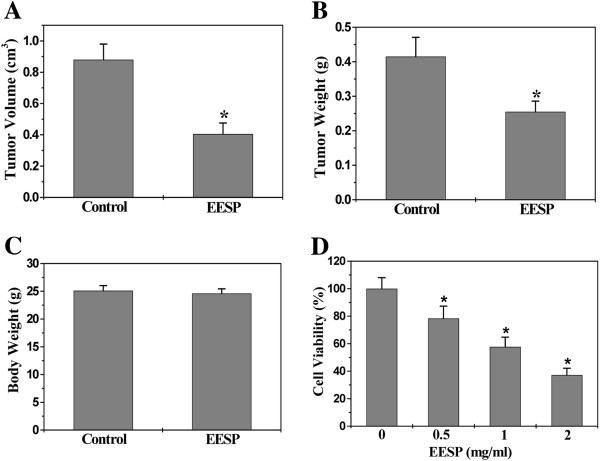
**Effect of EESP on tumor growth in colorectal cancer (CRC) xenograft mice and HT-29 colon cancer cells.** After tumor development, the mice were given intra-gastric administration of 6 g/kg/d dose of EESP or saline daily, 5 days per week for 16 days. Tumor volume (**A**), tumor weight (**B**) and body weight (**C**) were was measured at the end of experiment. (**D**) HT-29 cell viability was determined by the MTT assay after cells were treated with the indicated concentrations of EESP for 24 h. The data were normalized to the viability or survival of control cells (100%, treated with 0.5% DMSO vehicle). Data shown are averages with S.D. (error bars) from 9 individual mice in each group or from three independent cell-based experiments. *P< 0.01, versus controls.

The anti-tumor activity of EESP *in vivo* was determined through examination of the tumor weight and volume in CRC xenograft mice; and its adverse effects were evaluated by measuring the body weight changes. As shown in Figure 2A & B, EESP treatment significantly reduced both tumor volume and tumor weight, as compared to control (*P* < 0.01). However, EESP treatment had no effect on the changes of body weight (Figure 2C). Thus, it is suggested that EESP effectively suppresses CRC growth *in vivo*, without apparent signs of toxicity. To evaluate the *in vitro* anti-tumor activity of EESP, we performed MTT assay to examine its effect on the viability of human colon carcinoma HT-29 cells. As shown in Figure 2D, treatment with 0–2 mg/ml of EESP 24 h respectively reduced cell viability by 22-63%, compared to untreated control cells (*P* < 0.01), suggesting that EESP inhibits CRC cell growth *in vitro* in dose-dependent manners.

### EESP changes the histological structure of tumor in CRC xenograft mice

**Figure 3:**
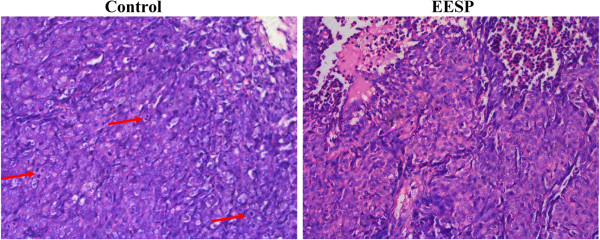
**Effect of EESP on the structural changes of tumor in CRC mice.** Tumor tissues were fixed with 10% buffered formalin for 24 h. Samples were then paraffin-embedded, sectioned, and stained with hematoxylin and eosin (H&E). Histopathological changes were observed under a light microscope. Images are representative photographs taken at a magnification of 200 ×.

The tumor histological changes in CRC mice were observed via light microscopy after HE staining. As shown in Figure 3, tumor cells were densely packed in tissues of the control group, but were sparse in tissues of EESP-treated group. There was marked cytonuclear pleomorphism, mitotic activity (red arrow). Large blood vessels and red blood cells could be observed in control group. In EESP group, portions of cells were necrotic and inflammatory cells were observed around the tumor tissue.

### EESP induces apoptosis, inhibits cancer cell proliferation and angiogenesis in CRC xenograft mice

**Figure 4:**
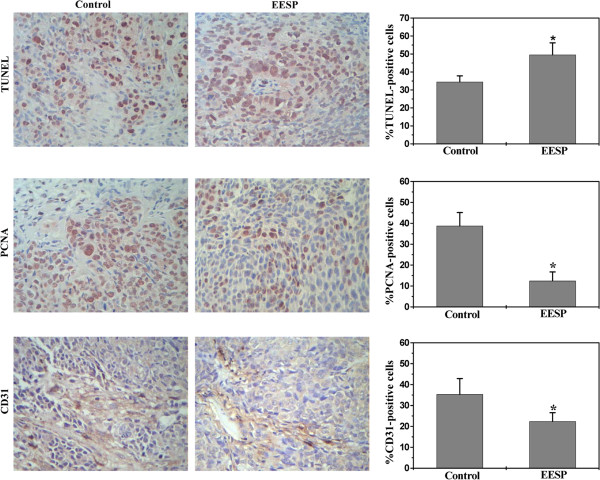
**Effect of EESP on cell apoptosis, proliferation and angiogenisis in CRC mice.** At the end of the experiment, tumor tissues from control and EESP-treated group were processed for immunohistochemical staining for TUNEL, PCNA or CD31. The photographs are representative images taken at a magnification of 400 ×. Quantification of immunohistochemical assay was represented as percentage of positively-stained cells. Data shown are averages with S.D. (error bars) from 9 individual mouse in each group. *P< 0.01, versus controls.

Cell apoptosis and proliferation in tumors from CRC xenograft mice was evaluated via immunohistochemical staining (IHS) for TUNEL and PCNA, and the intratumoral microvessel density (MVD) was examined by using IHS for the endothelial cell-specific marker CD31. As shown in Figure 4, the percentage of TUNEL-positive cells in EESP-treated or control mice was 49.6 ± 6.63% or 34.5 ± 3.34% (*P* < 0.01), respectively; whereas the proportion of cells expressing PCNA and CD31 was 12.49 ± 4.25% and 22.41 ± 4.11% in EESP-treated mice, 38.78 ± 5.09% and 35.34 ± 7.54% in controls. Collectively, these data suggest that EESP promotes colorectal cancer cell apoptosis, as well as inhibits cell proliferation and inhibits tumor angiogenisis.

### EESP suppresses STAT3 phosphoralytion in CRC xenograft mice

**Figure 5:**
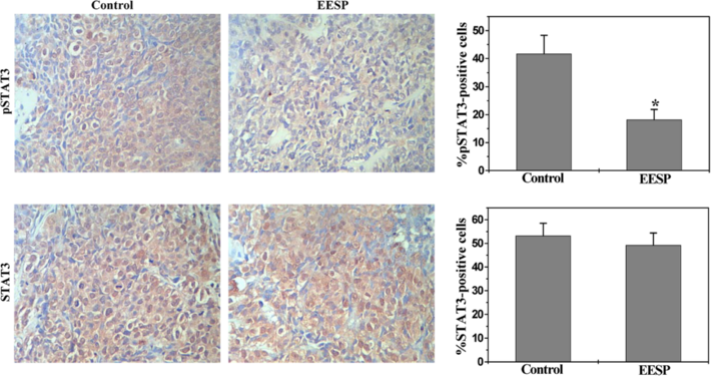
**Effect of EESP on phosphorylation of STAT3 in CRC mice.** At the end of the experiment, tumor tissues from control and EESP-treated group were processed for immunohistochemical staining for phosphorylated STAT3 (pSTAT3) or total STAT3. The photographs are representative images taken at a magnification of 400 ×. Quantification of immunohistochemical assay was represented as percentage of positively-stained cells. Data shown are averages with S.D. (error bars) from 9 individual mouse in each group. *P< 0.01, versus controls.

After activated via phosphorylation, STAT3 modulates the expression of key genes involved in the regulation of cell apoptosis, proliferation and angiogenesis; we therefore examined the effect of EESP on STAT3 phosphoralytion level (pSTAT3) using IHS assay in tumor tissues. Data from Figure 5 indicated that the percentage of pSTAT3-positive cells in control or EESP-treated mice was 41.71 ± 6.59% and 18.24 ± 3.63%, respectively (*P* < 0.01), suggesting that EESP treatment significantly suppresses the activation of STAT3 pathway in CRC mice.

### EESP regulates the expression of Bcl-2, Bax, Cyclin D1, CDK4, VEGF-A and VEGFR-2 in CRC xenograft mice

**Figure 6:**
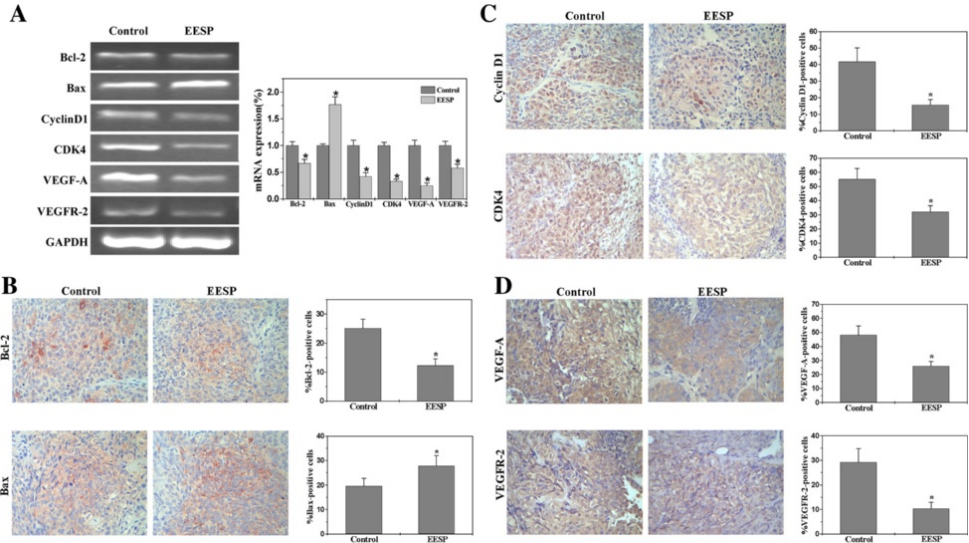
**Effect of EESP on the expression of Bcl-2, Bax, Cyclin D1, CDK4, VEGF-A and VEGFR-2 in CRC mice.** (**A**) The mRNA levels in tumor tissues from control and EESP-treated group were determined by RT-PCR. GAPDH was used as an internal control. The data of densitometric analysis were normalized to the mean mRNA expression of untreated control (100%). (**B-D**) The protein expression of Bcl-2, Bax, Cyclin D1, CDK4, VEGF-A and VEGFR-2 in tumor tissues was analyzed via immunohistochemical assay. The photographs are representative images taken at a magnification of 400 ×. Quantification of immunohistochemical assay was represented as percentage of positively-stained cells. Data shown are averages with S.D. (error bars) from 9 individual mouse in each group. *P< 0.01, versus controls.

To further explore the mechanism of EESP’s anti-tumor activity, we performed RT-PCR and IHS analyses to respectively examine the mRNA and protein expression of Bcl-2, Bax, Cyclin D1, CDK4, VEGF-A and VEGFR-2 in CRC mice. PCR data showed that EESP treatment significantly reduced the mRNA expression of pro-proliferative Cyclin D1 and CDK4, anti-apoptotic Bcl-2, pro-angiogenic VEGF-A and VEGFR-2 in CRC mice, whereas that of pro-apoptotic Bax was significantly increased after EESP treatment (Figure 6A). In addition, results of IHS assay showed that the protein expression patterns of these factors were similar to their respective mRNA levels (Figure 6B-D). The percentage of Bcl-2-, Bax-, Cyclin D1-, CDK4-, VEGF-A- or VEGFR-2- positive cells in control group was 25.1 ± 3.09%, 19.6 ± 3.17%, 41.85 ± 8.29% , 55.14 ± 7.57%, 48.01 ± 6.54% or 29.17 ± 5.57%, respectively, whereas that in EESP-treated mice was 12.37 ± 2.14%, 27.84 ± 4.13%, 15.71 ± 3.27%, 32.17 ± 4.3%, 26.14 ± 3.33% or 10.4 ± 2.54% (*P* < 0.01).

## Discussion

The development of cancer including colorectal cancer (CRC) is strongly associated with the dysregulation of multiple biological processes, such as imbalance between cell apoptosis and proliferation, and uncontrolled angiogenesis, which is mediated by aberrant activation of multiple intracellular signal transduction pathways. Given the complexity of cancer pathogenesis and progression, many of the currently used anti-tumor agents, which typically target a single intracellular pathway, might not always be effective on complex tumor systems but often generates drug resistance after long-term use. Additionally, most currently used chemotherapies possess intrinsic toxicity against normal cells. Thus, development of novel anti-cancer agents is needed. Natural products, such as traditional Chinese medicines (TCM), have been used clinically for thousands of years as important alternative remedies for a wide variety of diseases including cancer, without apparent adverse effects. Moreover, TCM are considered to be multi-component and multi-target agents exerting their therapeutic function in a more holistic way [35-38]. Therefore, discovering naturally occurring anti-tumor agents has received great interest. *Spica Prunellae* is a well-known Chinese medicinal herb with multiple pharmacological applications including anti-tumor property. The anti-cancer activity of *Spica Prunellae* was recorded in several ancient Chinese medical books, including < Treasury of Words on the Materia Medica > (‘Ben Cao Hui Yan’ in Chinese) written in Ming Dynasty, and < A Newly Revised Materia Medica > (‘Ben Cao Cong Xin’ in Chinese) written in Qing Dynasty. Indeed, *Spica Prunellae* has long been used as a major component in several TCM formulas for cancer treatment [39,40]. Although we previously demonstrated the anti-cancer activities of *Spica Prunellae in vitro*[33,34], the therapeutic efficacy against tumor growth *in vivo* and the underlying mechanism remain largely unclear. Using a CRC mouse xenograft model, in this study we found that the ethanol effect of *Spica Prunellae* (EESP) significantly reduced both tumor volume and tumor weight in CRC xenograft mice, whereas EESP treatment had no effect on the changes of body weight, demonstrating that *Spica Prunellae* is potent in suppressing CRC growth *in vivo*, without apparent signs of toxicity.

Cancer cell is characterized by imbalance between cell apoptosis and proliferation [41]. Mitochondria play an important role in this process of apoptosis, which is highly regulated by Bcl-2 family proteins including both anti-apoptotic members such as Bcl-2 and pro-apoptotic members such as Bax. The ratio of anti- and pro-apoptotic Bcl-2 family members determines the fate of cells, and alteration of the ratio by aberrant expression of these proteins impairs the normal apoptotic program contributing to various diseases including cancer. For instance, higher Bcl-2 to Bax ratios are commonly found in cancers, which not only confers a survival advantage to the cancer cells but also causes resistance to chemotherapies [42]. Eukaryotic cell proliferation is primarily regulated by cell cycle, wherein G1/S transition is a major checkpoint responsible for initiation and completion of DNA replication. G1/S progression is strongly regulated by the combined activity of the Cyclin D1/CDK4 complex [43]. An unchecked or hyperactivated Cyclin D1/CDK4 complex often leads to uncontrolled cell division and therefore cancers [44,45]. Therefore, re-balancing of cell apoptosis and proliferation via regulation of the expression of apoptosis- or cell cycle-related genes is a promising strategy for cancer chemotherapies. Using a CRC mouse xenograft model, we demonstrated that the ethanol effect of *Spica Prunellae* (EESP) inhibited cancer growth *in vivo*, without apparent toxicity. By using immunohistochemical staining for TUNEL and PCNA we found that EESP promoted apoptosis and inhibited proliferation in tumor tissues. In addition, the pro-apoptotic and anti-proliferative activities of EESP were mediated by its effects on the expression of relevant genes. EESP treatment profoundly increased the pro-apoptotic Bax/Bcl-2 ratio and down-regulated the expression of pro-proliferative Cyclin D1 and CDK4.

Angiogenesis, a process involving the growth of new blood vessels from the pre-existing vasculature, is essential for continued growth of the tumor and provides an avenue for hematogenous metastasis [46]. Vascular endothelial growth factor A (VEGF-A) is one of the most effective biologic inducers of angiogenesis, which is highly expressed in a wide variety of human cancers and is associated with cancer progression, invasion and metastasis, and poor patient prognosis. VEGF-A exerts its pro-angiogenic function via binding to its specific receptors including VEGFR-2 which is located on vascular endothelial cells. Binding of VEGF-A to VEGFR-2 leads to series of angiogenic processes [47]. Herein, we found that EESP significantly reduced the intratumoral microvessel density (MVD) in CRC xenograft mice via down-regulating the expression of VEGF-A and VEGFR-2, demonstrating its *in vivo* anti-angiogenic activity.

STAT3 is a crucial transcription factor that plays an important role in relaying extracellular signals initiated by cytokines and growth factors from the cytoplasm to the nucleus. Following activation via phosphorylation, STAT3 proteins dimerize and translocate to the nucleus where they regulate the expression of numerous critical genes involved in cell cycle progression, proliferation, and angiogenesis, including above-mentioned Bcl-2, Bax, Cyclin D1, CDK4 and VEGF-A. Therefore, suppression of STAT3 activation should be a promising approach in the development of anti-cancer therapies. Using immunohistochemical staining here we observed that EESP significantly reduced the phosphoralytion level of STAT3 in tumors of CRC mice, consistent with its pro-apoptotic, anti-proliferative and anti-angiogenic activities.

## Conclusions

In conclusion, for the first time we demonstrate that *Spica Prunellae* inhibits colorectal cancer growth *in vivo* via promoting the apoptosis of cancer cells, inhibition of proliferation and anti- angiogenesis, which is mediated by the suppression of the STAT3 pathway. Our findings suggest that *Spica Prunellae* may be a potential novel therapeutic agent for the treatment of cancers with constitutive activation of STAT3.

## Abbreviations

CRC: Colorectal cancer; STAT3: Signal transducer and activator of transcription 3; EESP: Ethanol extract of *Spica Prunellae*; TCM: Traditional Chinese medicine; TUNEL: Terminal deoxynucleotidyl transferase-mediated dUTP nick end labeling; PCNA: Proliferating cell nuclear antigen; CDK4: Cyclin dependent kinase 4; VEGF-A: Vascular Endothelial Growth Factor A.

## Competing interest

The authors declare no financial or commercial conflict of interest.

## Authors’ contributions

WL participated in the project design, carried out most of the experiments, contributed to the data interpretation, and wrote the initial drafts of the manuscript. LZ and QZ contributed to the *in vivo* tumor xenograft study and the *in vitro* MTT assay. JZ and ZC contributed to RT-PCR, immunohistochemistry assay and Western Blot. JZ and SL contributed to HPLC analysis. WX contributed to preparation of ethanol extract from *Spica Prunellae*. JP contributed to the conception and design of the entire study and the final editing of the manuscript. All authors have read and approved the manuscript for publication.

## Pre-publication history

The pre-publication history for this paper can be accessed here:

http://www.biomedcentral.com/1472-6882/13/144/prepub
